# Using Machine Learning to Create Prognostic Systems for Primary Prostate Cancer

**DOI:** 10.3390/diagnostics15192462

**Published:** 2025-09-26

**Authors:** Kevin Guan, Andy Guan, Anwar E. Ahmed, Andrew J. Waters, Shyh-Han Tan, Dechang Chen

**Affiliations:** 1 F. Edward Hébert School of Medicine, Uniformed Services University of the Health Sciences (USUHS), Bethesda, MD 20814, USAanwar.ahmed@usuhs.edu (A.E.A.); andrew.waters@usuhs.edu (A.J.W.); 2 The Henry M. Jackson Foundation for the Advancement of Military Medicine Inc., Bethesda, MD 20817, USA; 3 Commissioned Corps of the U.S. Public Health Service (USPHS), Rockville, MD 20852, USA; 4 Center for Prostate Disease Research (CPDR), Murtha Cancer Center Research Program, Department of Surgery, Uniformed Services University of the Health Sciences, Bethesda, MD 20817, USA; 5 Genitourinary Malignancies Branch, Center for Cancer Research, National Cancer Institute, Bethesda, MD 20814, USA

**Keywords:** prostate cancer, cancer staging, machine learning, EACCD, dendrogram, C-index, survival curves

## Abstract

**Background:** Cancer staging, guided by anatomical and clinicopathologic factors, is essential for determining treatment strategies and patient prognosis. The current gold standard for prostate cancer is the American Joint Committee on Cancer (AJCC) Tumor, Lymph Node, and Metastasis (TNM) Staging System 9th Version (2024). This system incorporates five prognostic variables: tumor (T), spread to lymph nodes (N), metastasis (M), prostate-specific antigen (PSA) levels (P), and Grade Group/Gleason score (G). While effective, further refinement of prognostic systems may improve prediction of patient outcomes and support more individualized treatment. **Methods:** We applied the Ensemble Algorithm for Clustering Cancer Data (EACCD), an unsupervised machine learning approach. EACCD involves three steps: calculating initial dissimilarities, performing ensemble learning, and conducting hierarchical clustering. We first developed an EACCD model using the five AJCC variables (T, N, M, P, G). The model was then expanded to include two additional factors, age (A) and race (R). Prostate cancer patient data were obtained from the Surveillance, Epidemiology, and End Results (SEER) program from the National Cancer Institute. **Results:** The EACCD algorithm effectively stratified patients into distinct prognostic groups, each with well-separated survival curves. The five-variable model achieved a concordance index (C-index) of 0.8293 (95% CI: 0.8245–0.8341), while the seven-variable model, including age and race, improved performance to 0.8504 (95% CI: 0.8461–0.8547). Both outperformed the AJCC TNM system, which had a C-index of 0.7676 (95% CI: 0.7622–0.7731). **Conclusions:** EACCD provides a refined prognostic framework for primary localized prostate cancer, demonstrating superior accuracy over the AJCC staging system. With further validation in independent cohorts, EACCD could enhance risk stratification and support precision oncology.

## 1. Introduction

Prostate cancer is the most prevalent cancer among American men, with an estimated 299,010 new diagnoses and 35,250 deaths in 2024 [1]. Men of African ancestry are disproportionately affected, with higher rates of both diagnosis and mortality compared to men of European ancestry [1]. The widespread use of prostate-specific antigen (PSA) testing and advances in treatment has helped to reduce prostate cancer mortality by half since its peak in 1993 [2,3,4]. However, the potential for false positives has led to debates over PSA testing [5]. Meanwhile, the US Preventive Services Task Force (USPSTF) recommendations against screening in certain age groups have contributed to an increase in both localized and distant-stage diseases [6,7]. Accurately staging the disease is crucial for effective treatment and improved survival, as the 5-year survival rate drops significantly from 100% for localized cancer to 33% for distant stage patients [1,8].

The current standard for prostate cancer staging is the American Joint Committee on Cancer (AJCC) Tumor, Lymph Node, and Metastasis (TNM) Staging System [9,10]. This system relies on five key clinical–pathologic factors: the tumor size and extent (T), spread to lymph nodes (N), presence of metastasis (M), PSA levels (P), and Grade Group (G) [9,11]. The AJCC TNM system classifies prostate cancer into four stages [9]. Stage I is a non-palpable tumor that is undetectable by digital rectal exam (DRE) or imaging. In Stage II, the tumor is larger and detectable within the prostate by DRE or imaging. Stage III tumors have spread to nearby tissues such as seminal vesicles, while Stage IV indicates the tumor has metastasized to distant parts of the body, such as lymph nodes or bones. Localized prostate cancer (stages I, II, and IIIA) and regional prostate cancer metastasized to lymph nodes (stages IIIB and IVA) have an almost 99% five year survival rate, and distant prostate cancer (stage IVB) and unknown or unstaged prostate cancer have a five year relative survival rate of 34% and 92%, respectively [8]. Though staging is important to guide treatment modalities, the AJCC TNM System has significant limitations. It omits potentially important variables and has shown limited accuracy in predicting progression and recurrence. As demonstrated by the current AJCC standards, there is illogical staging of localized prostate cancer as stage IIIA along with the other localized prostate cancer stages I and II. The stage IIIA discrepancy is further highlighted as stage IIIA is treated as localized prostate cancer in contrast to stage IIIB, as regional prostate cancer. Other studies demonstrate the difficulty in distinguishing survival outcomes between stage III and stage IV patients [12]. Attempts to revise or improve the AJCC staging have been limited. For example, Sun et al. [12] proposed a modified staging system based on a nomogram that integrates T, N, and M stages, primary and secondary Gleason pattern scores, and PSA levels. However, nomograms rely on the proportional hazards assumption, which is often violated in real-world datasets, limiting their prognostic accuracy. In addition, there are no standardized guidelines for translating nomogram scores into discrete risk groups, reducing their practical utility.

To overcome these limitations, we applied the Ensemble Algorithm for Clustering Cancer Data (EACCD) [13,14] to develop a more accurate prognostic system. This machine learning algorithm has been successfully used to cluster survival data to identify highly accurate prognostic groups from survival data for other cancers, including breast [15,16], colorectal [17], and lung cancer [18], as well as in ovarian [19] and endometrial cancers [20], Hodgkin and Non-Hodgkin lymphoma [21], and melanomas [22]. By using EACCD on population-level SEER data, we aim to enhance prostate cancer staging and provide a more accurate tool for predicting patient outcomes. We first constructed models using the five standard AJCC variables and then extended them by incorporating age and race, two well-established prognostic factors not currently included in the AJCC system. We subsequently evaluated the performance of the EACCD models using the validation dataset.

This paper is organized as follows: Section 2 describes the methods, Section 3 presents the results, and Section 4 provides the conclusions.

## 2. Methods

### 2.1. Clinical–Pathologic Datasets

Prostate cancer data as identified by the International Classification of Disease for Oncology (Third Edition) [ICD-O-3] histology code (C61.9) were extracted from de-identified data on patient demographics from the National Institutes of Health (NIH) National Cancer Institute (NCI) Surveillance, Epidemiology, and End Results Program (SEER) database. A total of 254,390 out of the total 354,781 prostate patients diagnosed between 2010 and 2015 were retrieved from 17 datasets from the SEER 17 Registries Database 2023 version [23]. SEER cases before 2010 were excluded to reflect the same revision criteria as AJCC’s TNM Staging Editions used to diagnose prostate cancers on or after 1 January 2020, with the adoption of the PSA and Grade Group. Cases after 2015 were also excluded because 2015 was the last year that allowed a follow up year of at least five years. For the study data, survival time measured in months were included in the prostate-specific survival analysis.

Following the AJCC staging system, this study included five primary variables: Tumor (T), Lymph Node (N), Metastasis (M), PSA (P), and Grade Group (G). Additionally, two other variables—Age (A) and Race (R)—were included due to their established prognostic influence [24,25,26,27]. A detailed description of these seven variables is provided in Table 1. The original AJCC staging system is outlined in Table 2, compiled from information in the literature [9,10].

Other variables used in clinical prognosis such as androgen deprivation therapy, neoadjuvant androgen deprivation therapy, and genetic predisposition to prostate cancer from family lineage were not collected in the SEER database. Therefore, they could not be included in the EACCD calculations.

**Figure 1:**
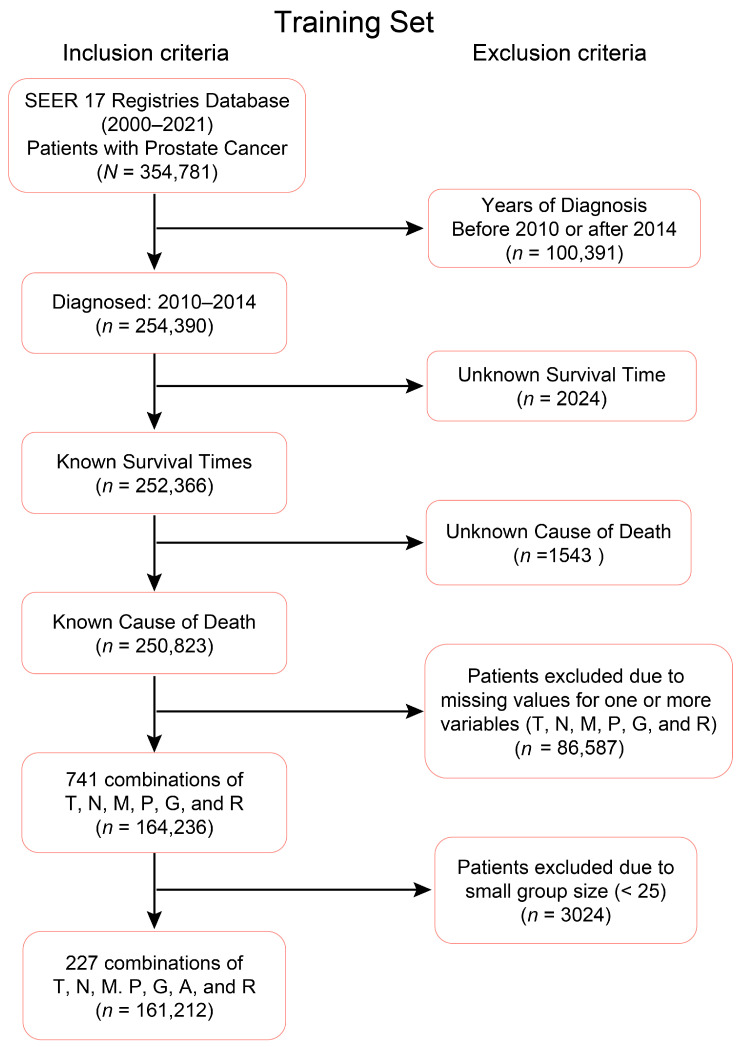
Criteria for the training dataset. Out of the 354,781 prostate cancer patients diagnosed and recorded in 2010–2021 from the SEER database, the 254,390 cases diagnosed from 2010–2014 inclusive were wrangled to form the initial dataset. Out of the 254,390 cases from the years 2010–2014, there are 164,236 cases with known survival time and known cause of death that form 741 known combinations of levels of (T)umor, Lymph (N)ode, (M)etastasis, (P)SA, (G)rade Group, (A)ge, and (R)ace. The final training set consists of 227 combinations each containing at least 25 patients.

**Figure 2:**
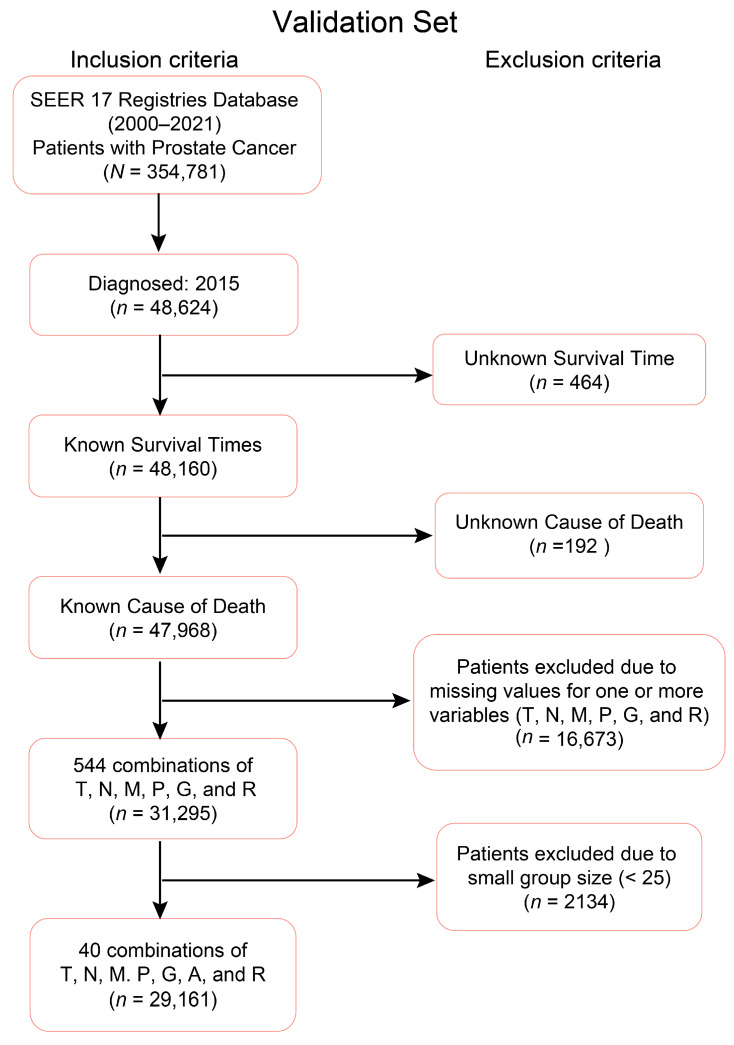
Criteria for the validation dataset. Out of the 48,624 prostate cancer patients diagnosed and recorded in 2015 from the NIH SEER database, there are 31,295 cases with known survival time and known cause of death that form 544 known combinations of levels of (T)umor, Lymph (N)ode, (M)etastasis, (P)SA, (G)rade Group, (A)ge, and (R)ace. The validation set consists of 40 combinations each containing at least 25 patients.

Cases diagnosed between 2010 and 2014 were used to create the training dataset, while cases diagnosed in 2015 served as the validation set. The inclusion and exclusion criteria outlined in Figure 1 and Figure 2 were applied to filter the training and validation datasets, respectively. The final training and validation datasets contain known values corresponding to the prognostic variables of interest. Any missing or unknown values were not imputed, and therefore excluded. Only patients with known Survival Time and Cause of Death were included because these variables are essential for reliable survival analysis; without them, it is not possible to calculate time-to-event outcomes or to distinguish prostate cancer–specific mortality. In the datasets, these exclusions represented a small fraction of the overall cohort (e.g., 464 patients, 1.6% of the total), and their removal ensured that survival endpoints were well-defined for all included cases. Any combination during the data processing step that had less than 25 patients was also excluded due to the low statistical power associated with lower representation. In this context, a “combination” refers to a subset of patients corresponding to specific levels of the selected factors, such as T4N1M1, which represents patients with T = T4, N = N1, and M = M1. A threshold of 25 was used to optimize the robustness of the statistical techniques, though any reasonable cutoff could be applied. Importantly, after applying these exclusion principles, the demographic and clinical–pathologic distribution of the final dataset remained consistent with the broader SEER prostate cancer population, suggesting no systematic bias was introduced. Thus, the final dataset structure was robust, reproducible, and appropriate for developing and validating prognostic models. The sample characteristics distribution of the variables in the training and validation datasets are outlined in Table 3 and Table 4, respectively. The EACCD algorithm was utilized on the training data to develop prognostic models, which were subsequently validated using the validation dataset.

### 2.2. EACCD

The Ensemble Algorithm for Clustering Cancer Data (EACCD) is a machine learning technique introduced by Chen et al. [13,16], designed to partition cancer survival data based on combinations of factors. Given a collection of combinations $\left\{C_{1},C_{2},...,C_{n}\right\}$ and nonnegative weights $w_{1},w_{2},...,w_{n}\;$ with $\sum_{k=1}^{n}w_{k}=1$, the EACCD consists of the following three main steps (see Section *Experiments* in [14]):

1. Define the initial dissimilarity $dis_{0}(C_{i},C_{j})$ for any pair $C_{i}$ and $C_{j}$.
2. For each k with 1≤ k≤n, apply the two-phase Partitioning Around Medoids and the initial dissimilarities in Step 1 to partition combinations into k clusters. $\delta_{k}(i,j)$ is defined as 1 if $C_{i}$ and $C_{j}$ are not assigned into the same cluster, otherwise $\delta_{k}(i,j)$ was defined as 0. Then the learned dissimilarity is defined as follows: $dis(C_{i},C_{j})=\sum_{k=1}^{n}w_{k}\delta_{k}(i,j)$.
3. Perform hierarchical clustering to cluster the combinations by using $dis(C_{i},C_{j})$.

Each of these steps can employ different approaches. In the present study, the initial dissimilarity between $C_{i}$ and $C_{j}$ is defined as the survival difference between them, estimated using the effect size derived from the Gehan–Wilcoxon test statistic [14,28]. The ensemble learning process utilizes weights $w_{k}=1/n$ for k=1,2,...,n. For hierarchical clustering, the minimax linkage method [29] is selected.

The EACCD algorithm generates a dendrogram showcasing all the possible combinations in terms of survival. Dendrograms enable researchers and physicians to understand the rationale behind the grouping of patients. Combinations can be partitioned by horizontally cutting the dendrogram to the specific level of dissimilarity. Harrell’s C-index (also known as the concordance index) [30] is then computed from the available (prognostic) groups of combinations to highlight the statistical predictive accuracy. Generally, the curve for the C-index plotted against the number of groups increases for a relatively small number of groups before plateauing as more groups are generated. The optimal n* of prognostic groups is obtained from the “knee” point of the C-index curve.

We conclude this subsection with the following note: When we focus on patient features and survival times (numerical data), EACCD serves as an outcome-guided clustering method, that is, a form of supervised clustering where the supervision is provided by the outcome of interest. In contrast, when we focus on the survival curves of combinations (curves treated as geometric objects rather than raw numbers), EACCD functions as a fully unsupervised learning method, clustering these unlabeled curves based solely on their shapes.

### 2.3. Prognostic Systems

The proposed EACCD was trained on SEER 2010–2014 data to obtain n* prognostic groups. Survival curves for these groups were then plotted with Kaplan–Meier estimates to visually examine the survival differences among the different prognostic groups. The final prognostic system, named the EACCD prognostic system, consists of the dendrogram, group assignment, C-index, and survival curves.

As more variables are included, the number of possible combinations of values of variables increases, making it more difficult to visually examine the dendrogram. To address this, the dendrogram and corresponding group assignments can be summarized in tables (see the Results section below). In the EACCD system, risk stratification can be evaluated by visually examining the survival curves of the prognostic groups, while the accuracy of survival prediction is measured using the C-index. Overall, the EACCD system has been shown to provide effective patient risk stratification and high predictive accuracy.

The use of the EACCD system is straightforward. First, a patient’s values for the relevant variables are determined. Next, the patient is assigned to a prognostic group based on these values. Finally, the system’s survival curve associated with that group is used to help doctors and the patient understand the prognosis and guide treatment decisions.

### 2.4. Software

All statistical analyses as well as their respective visualizations were conducted in R (Version 4.2.2) using the following libraries: *survival* (Version 3.4-0), *cluster* (Version 2.1.4), *protoclust* (Version 1.6.4), *factoextra* (Version 1.0.7), and *compareC* (Version 1.3.2).

## 3. Results

In this section, we begin by presenting the AJCC 9th edition staging system and the EACCD system, both of which are based on five key variables: tumor, lymph nodes, metastasis, PSA, and Grade Group. Then an extended version of the EACCD system, which includes two additional variables race and age, was introduced. Finally, the validation results were shown to demonstrate reproducibility. For simplicity, the various AJCC prognostic stages are referred to as stages, while the corresponding EACCD outputs are referred to as groups.

### 3.1. Five Variable AJCC System

**Figure 3:**
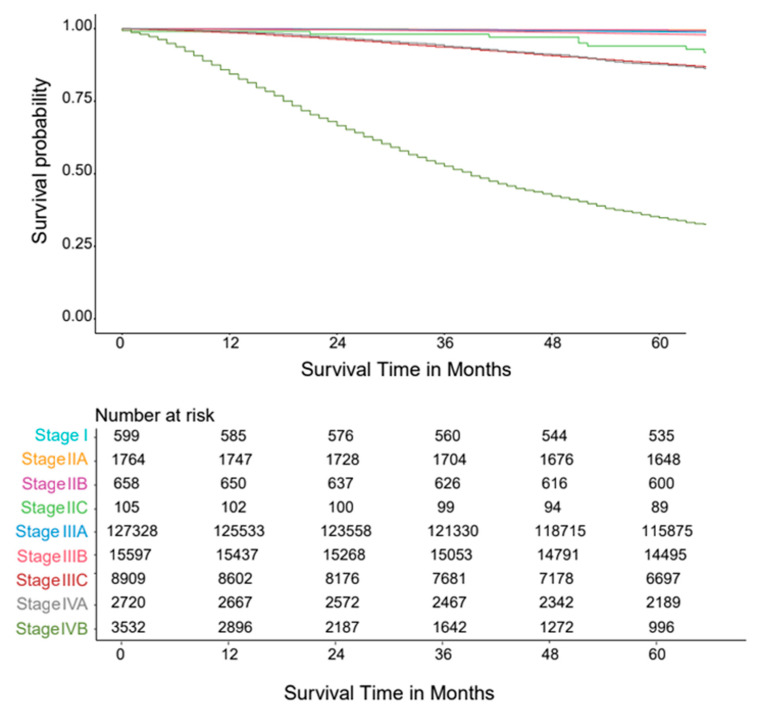
Survival curves plot using the training data and the AJCC 9th version prostate cancer staging system.

The original stages from the AJCC TNM System 9th Version consisting of four principal stages (I, II, III, and IV) from the five variables of Tumor (T), Metastasis (M), Lymph Nodes (N), PSA(P), and Grade Group (G) were included for comparisons to the groupings produced by the EACCD algorithm. Depending on the anatomic extent of the disease, PSA levels, and Grade Group, the AJCC TNM Prostate Staging System can be further divided into nine prognostic substages (I, IIA, IIB, IIC, IIIA, IIIB, IIIC, IVA, and IVB) as described in Table 2. Survival curves provide visual comparisons of the substages over time within the data. By applying the AJCC 9th Version Staging System to the training dataset, the survival curves shown in Figure 3 were generated. These curves reveal weak separations between stages. For instance, the curve for Stage I closely resembles that of Stage IIA, and the curve for Stage IIC is close to that of Stage IIIA. The C-index for the AJCC 9th Version was calculated to be 0.7676 (95% CI: 0.7622–0.7731).

### 3.2. Five Variable EACCD System

**Figure 4:**
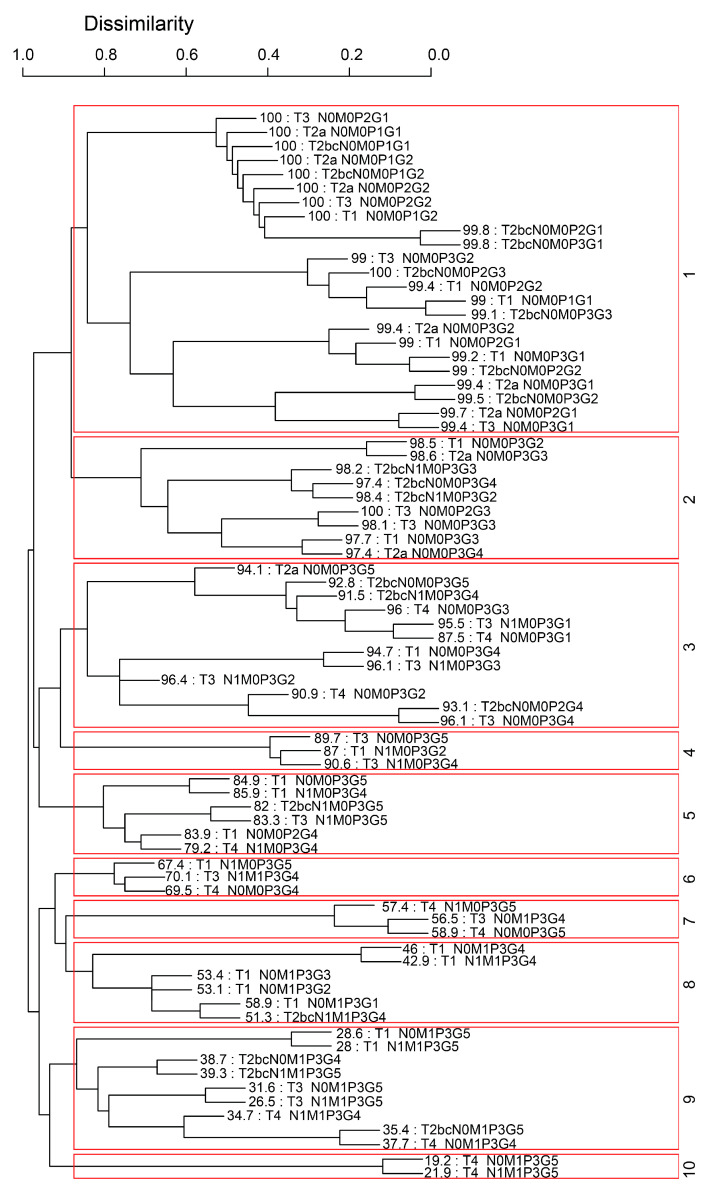
Dendrogram generated by the EACCD using five variables: T, N, M, P, and G.

**Figure 5:**
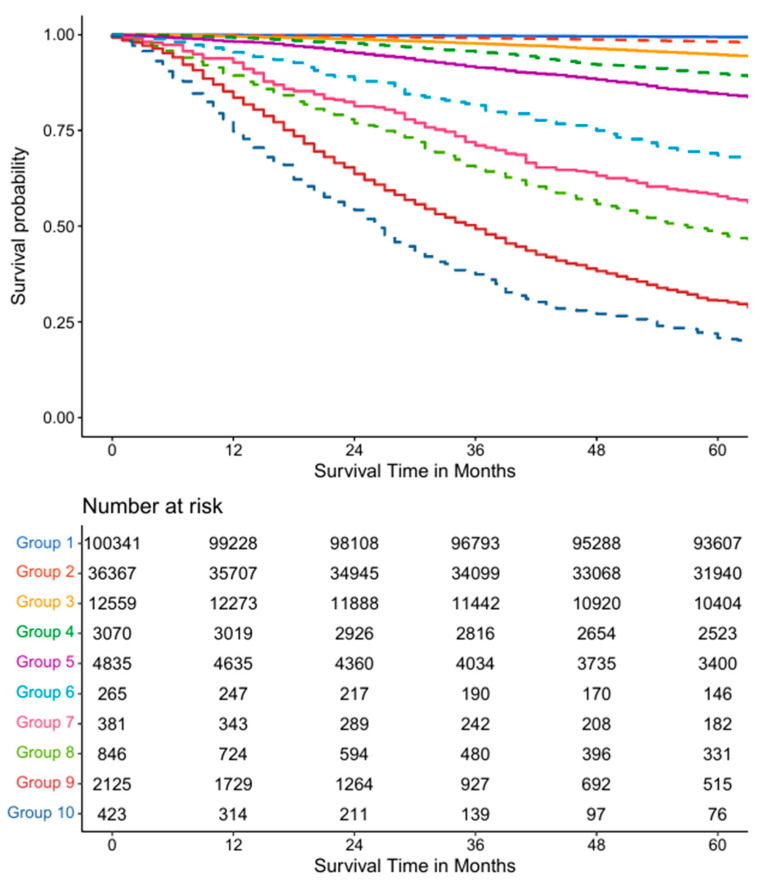
Survival curves of the 10 groups as extrapolated from Figure 4.

**Figure 6:**
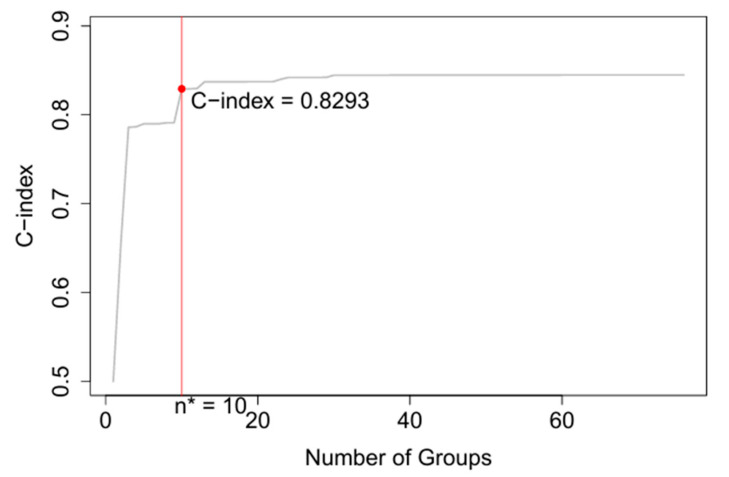
The C-index plot generated using five variables: T, N, M, P, and G. Here n* (10) groups correspond to a C-index of 0.8293.

Using the same training data and the same five variables—T, N, M, P, and G—as in the AJCC system, the EACCD algorithm clustered 227 unique combinations of patients into 10 distinct groups in Figure 4. To aid readability, Supplementary Table S1 lists the specific combinations for each of the 10 groups in Figure 4. In addition, Supplementary Table S2 shows the number of events (deaths) in each of the 10 groups. The survival curves for these 10 groups were plotted in Figure 5. Note that the separation between any two adjacent curves is statistically significant (log-rank test *p* < 0.05). Comparing Figure 5 with Figure 3 shows that EACCD distinguishes and stratifies prostate cancer patients much more effectively than the AJCC system. Furthermore, the C-index for EACCD on the training set is 0.8293 (95% CI: 0.8245–0.8341) as demonstrated in Figure 6, which is higher than the AJCC C-index of 0.7676 (95% CI: 0.7622–0.7731).

### 3.3. Seven Variable EACCD System

**Figure 7:**
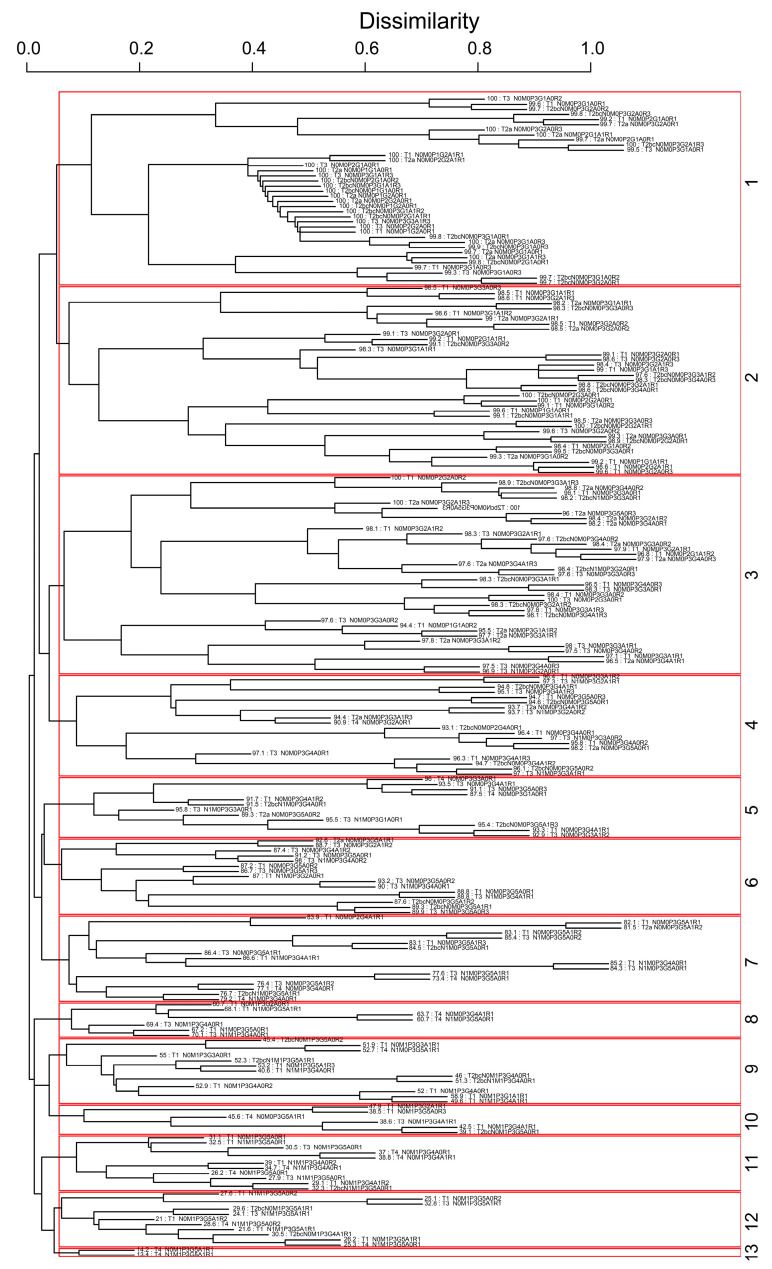
Dendrogram generated by the EACCD using seven variables: T, N, M, P, G, A, and R.

**Figure 8:**
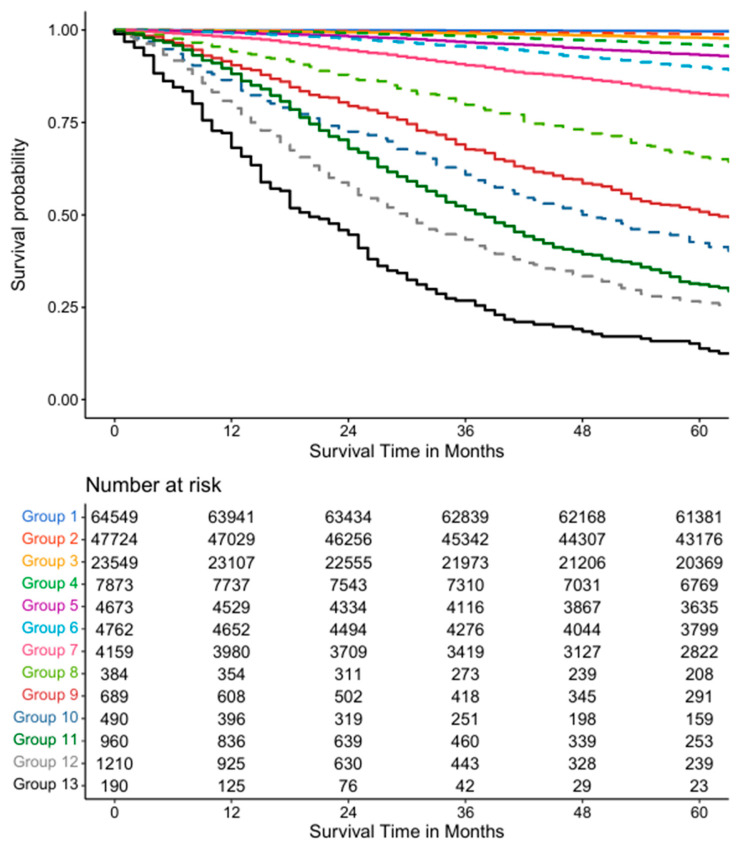
Survival Curves of the 13 groups as extrapolated from Figure 7.

**Figure 9:**
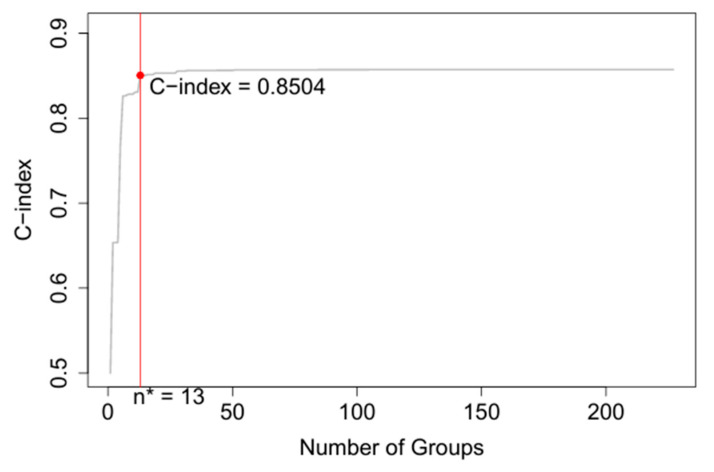
The C-index plot generated using seven variables: T, N, M, P, G, A, and R. Here n* (13) groups correspond to a C-index of 0.8504.

The inclusion of two additional variables, age and race, in the EACCD algorithm results in the dendrogram shown in Figure 7. To improve readability, Supplementary Table S3 lists the specific combinations corresponding to each of the 13 groups in Figure 7. Supplementary Table S4 further provides the number of events (deaths) within each group. The survival curves for the 13 resulting groups are shown in Figure 8. These curves are clearly separated (log-rank test *p* < 0.05 for any two adjacent curve comparisons), similar to those of the EACCD five-variable system in Figure 5. The C-index for this extended model is 0.8504 (95% CI: 0.8461–0.8547) as shown in Figure 9, which is higher than the AJCC C-index of 0.7676 (95% CI: 0.7622–0.7731) and the C-index of 0.8293 (95% CI: 0.8245–0.8341) from the EACCD five-variable model.

### 3.4. Validation

In Section 3.2 and Section 3.3, we developed the EACCD systems using five and seven variables with the training data. Both the five- and seven-variable EACCD systems outperformed the AJCC 9th Edition, providing clearer separation of survival groups and higher predictive accuracy. In this subsection, we present the results of evaluating these systems using the validation dataset. The validation process was as follows: we first applied the assignment rules of the systems to the validation dataset to group (“stage”) the patients. Next, we computed the C-indices and plotted survival curves based on these groups. Finally, we compared the C-indices and survival curves from the validation dataset with those from the training dataset in Section 3.2 and Section 3.3. Consistency of C-indices and survival curves between training and validation data indicates the reliability of the EACCD systems.

Table 5 compares the C-indices between the training and validation datasets in the five variable system and seven variable system. The five-variable EACCD system has a C-index of 0.8293 (95% CI: 0.8245–0.8341) in the training data and 0.8437 (95% CI: 0.8308–0.8566) in the validation data, while the seven-variable EACCD system has a C-index of 0.8504 (95% CI: 0.8461–0.8547) in the training data and 0.8585 (95% CI: 0.8468–0.8703) in the validation data. This indicates that the survival prediction performance of each EACCD system is consistent across both the training and validation datasets.

**Figure 10:**
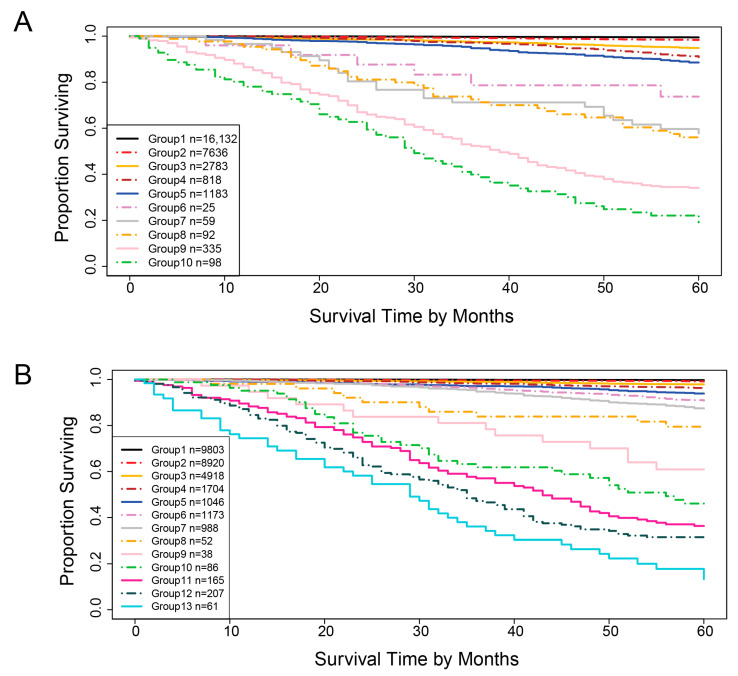
Survival curves for the validation dataset. (**A**) Five-variable system; (**B**) Seven-variable system.

Figure 10 shows the survival curves of the groups in the validation dataset. For the five-variable system, 10 survival curves corresponding to 10 groups are obtained. The separation between adjacent curves is significant (log-rank test *p* < 0.05) except for group 5 vs. group 4, group 7 vs. group 6, and group 8 vs. group 7. In these cases, the hazard ratios still point in the expected direction, providing objective evidence of group separation. Thus, applying the EACCD TNM system to the validation data yields 10 groups with clearly separated survival curves. Similarly, applying the EACCD TNMA system to the validation data results in 13 groups with separated survival curves. Here, the separation is significant for all adjacent pairs except group 10 vs. group 9, group 11 vs. group 10, and group 12 vs. group 11. Again, the hazard ratios for these comparisons remain consistent with the intended risk ordering. Together, these results indicate that the risk stratification performance of both systems is consistent across the training and validation datasets.

The above validation results suggest that EACCD performance is reproducible on future data. This further supports the robustness of the system for clinical application.

## 4. Discussion

There appear to be very few studies in the literature focused on developing methods to improve prostate cancer staging. A recent study by Sun et al. [12] modified the AJCC staging system using nomogram scores derived from multivariate Cox survival modeling. Sun et al. used a filtered dataset of 16,803 patients and achieved a C-index of 0.789 (95% CI: 0.777–0.801) compared to the calculated C-index 0.762 (95% CI: 0.748–0.776) on their dataset using the gold standard AJCC model [12]. In contrast, our EACCD model was trained on a larger and more recent dataset of 161,212 patients and produced C-indices of 0.8293 (95% CI: 0.8245–0.8341) and 0.8504 (95% CI: 0.8461–0.8547) for the five- and seven-factor prognostic systems, respectively.

The strength of unsupervised clustering algorithms lies in their ability to autonomously identify novel subgroups within complex and high-dimensional datasets. Traditional supervised methods, while effective for specific tasks like predicting biochemical recurrence, as seen in studies by Tan et al. [31] and Ekşi et al. [32], are ultimately designed to reinforce existing knowledge. Semwal et al. trained multiple supervised models to predict pathological stages using an XGBoost model that outperformed existing clinical methods [33]. Martelin et al. also used a gradient boost model that included PSA and personal variables to improve the model’s accuracy [34]. Nayan et al. employed an XGB algorithm to predict 5-year survival, highlighting the need to consider race in ML models to address healthcare disparities [35]. Finally, Fonseca et al. developed an XGB-based tool to predict whether there was enough circulating tumor DNA for informative genotyping in metastatic cases, further demonstrating the potential of machine learning to optimize biomarker use and refine risk stratification [36].

Our EACCD approach, which utilizes unsupervised clustering at its core, allowed us to identify new patient subgroups that were not evident in the current staging system. This is a crucial step toward creating more precise and personalized prognostic systems. Unsupervised learning approaches, with their ability to uncover hidden structures and inherent data patterns, provide a powerful tool for deepening our understanding of prostate cancer heterogeneity. They present an improvement beyond traditional approaches that rely on clinical pathologic features such as Gleason score, which often fail to capture the full picture of disease heterogeneity [37]. These approaches have been applied on diverse data types, including genomic and proteomic profiles, to discover new molecular subtypes with distinct clinical outcomes [38]. Increasingly, more studies employ integrated multi-modal systems that combine data from multiple sources such as digital pathology and clinical records to create highly accurate patient specific models [39]. These unsupervised clustering approaches provide more accurate and reliable prognostic information that is independent of existing risk classifications, therefore aiding in identifying at-risk patients who might otherwise be miscategorized. They will also improve as more data becomes available, supporting their value as a risk stratification tool to optimize for follow-up and reduce over-treatment.

The development of the EACCD prognostic system marks a promising step toward advancing personalized medicine in prostate cancer. Moving beyond the traditional and subjective committee-based decision-making, our EACCD method provides an algorithmic and data-driven framework. The EACCD system can be continually updated with new prognostic factors as they are discovered, such as circulating tumor DNA (ctDNA) and other clinical variables like lactate dehydrogenase [36]. This adaptability ensures its long-term relevance and accuracy. The potential for developing a clinical decision support tool that leverages EACCD to provide real-time prognostic groups for patients could significantly enhance clinical decision-making, optimize treatment strategies, and ultimately improve health outcomes.

## Figures and Tables

**Table 1:** Seven prognostic variables as defined by their usage in EACCD and AJCC, with information compiled from the American College of Surgeons (ACS) and the American Joint Committee on Cancer (AJCC) [9,10].

Variables	Levels	Definition/Description
Tumor stage (T)	Tx	Tumor was not evaluated
	T0	No evidence of the primary tumor
	T1	Tumor was not detected during Digital Rectal Exam (DRE) and not seen in imaging
	T2	Tumor is detected in DRE but locally confined in the prostate T2a: Tumor is half or less than one side of the prostate lobe T2b: Tumor is in more than ½ of the prostate lobe but not in the other lobe T2c: Tumor is present in both lobes
	T3	Tumor extends outside of the prostate T3a: Tumor extends outside of the prostate on one or both sides T3b: Tumor has spread to the seminal vesicles
	T4	Tumor has spread to nearby tissues outside of the prostate and seminal vesicles.
Metastasis to nearby or regional lymph nodes (N)	Nx	Nearby lymph nodes are not evaluated
	N0	No cancer cells are found in the lymph nodes
	N1	Cancer cells are found in the lymph nodes
Distant metastasis (M)	M0	Cancer has not spread past the prostate
	M1	Cancer has spread past the prostate M1a: Cancer has spread to the distant lymph nodes M1b: Cancer has spread to bones M1c: Cancer has spread to other organs and sites with or without bone disease
PSA (ng/mL)	P1	P < 10
	P2	10 ≤ P < 20
	P3	P ≥ 20
Grade Group (G) /Gleason Score (GS)	G1: GS ≤ 6	Only individual discrete well-formed glands
	G2: GS 7 (3 + 4)	Predominantly well-formed glands with lesser components of poorly formed, fused cribriform glands
	G3: GS 7 (4 + 3)	Predominantly poorly formed, fused, cribriform glands with lesser components of well-formed glands
	G4: GS 8	Only poorly formed, fused, cribriform glands or predominantly well-formed glands with a lesser component lacking or predominately lacking glands with a lesser component of well-formed glands
	G5: GS 9–10	Lacks gland formation (or with necrosis) with or without poorly formed/fused/cribriform gland
Age (years)	A0	<70
	A1	≥70
Race	R1	White, Caucasian, or European Ancestry
	R2	Black or African Ancestry
	R3	Other

**Table 2:** The AJCC TNM staging of prostate cancer. The AJCC 8th Edition was published in 2017, and the revision in the 2024 manual updated the naming of the 8th Edition to the 9th Version. The AJCC 9th Version (2024) has no changes to the prognostic prostate staging system compared with the 8th Edition. Data compiled from information in [9,10].

AJCC TNM Prostate Cancer Stages	Stage Subgroups	Grade Group	Tumor	Lymph Node	Metastasis	PSA
Stage I (Localized) Cancer is small and only in the prostate	I	1	cT1a–c cT2a pT2	N0	M0	<10
Stage II (Localized) Cancer is larger and may be in both prostate lobes but still confined in the prostate	IIA	1	cT1–ac cT2a pT2	N0	M0	10–20
	IIA	1	cT2b–c	N0	M0	10–20
	IIB	2	T1–2	N0	M0	<20
	IIC	3 4	T1–2	N0	M0	<20
Stage III (Locally Advanced) Cancer has spread from the prostate to nearby lymph nodes or seminal vesicles	IIIA	1–4	T1–2	N0	M0	≥20
	IIIB	1–4	T3–4	N0	M0	Any
	IIIC	5	Any T	N0	M0	Any
Stage IV (Metastatic/Advanced) Cancer has spread to other parts of the body such as bones, liver, or lungs.	IVA	Any	Any T	N1	M0	Any
	IVB	Any	Any T	Any N	M1	Any

**Table 3:** Sample characteristics of variable distributions in the Training Dataset.

Variables in the Training Dataset (*N* = 161, 212)	*n*
Tumor stage (T)	
T1	80,384 (50%)
T2a	12,897 (8.0%)
T2bc	46,316 (29%)
T3	20,599 (13%)
T4	1016 (0.6%)
Lymph node metastasis (N)	
N0	157,398 (98%)
N1	3814 (2.4%)
Metastasis (M)	
M0	157,680 (98%)
M1	3532 (2.2%)
Prostate-Specific Antigen (P)	
P1	926 (0.6%)
P2	2311 (1.4%)
P3	157,975 (98%)
Grade Group (G)	
G1	70,901 (44%)
G2	42,718 (26%)
G3	19,395 (12%)
G4	15,823 (9.8%)
G5	12,375 (7.7%)
Age (A)	
A0	115,078 (71%)
A1	46,134 (29%)
Race (R)	
R1	127,971 (79%)
R2	24,701 (15%)
R3	8540 (5.3%)

**Table 4:** Sample characteristics of variable distributions in the Validation Dataset.

Variables in the Validation Dataset (*N* = 29, 161)	*n*
Tumor stage (T)	
T1	15,360 (53%)
T2a	1801 (6.2%)
T2bc	7229 (25%)
T3	4614 (16%)
T4	157 (0.5%)
Lymph node metastasis (N)	
N0	28,293 (97%)
N1	868 (3.0%)
Metastasis (M)	
M0	28,636 (98%)
M1	525 (1.8%)
Prostate-Specific Antigen (P)	
P1	106 (0.4%)
P2	262 (0.9%)
P3	28,793 (99%)
Grade Group (G)	
G1	10,443 (36%)
G2	8357 (29%)
G3	4253 (15%)
G4	3252 (11%)
G5	2856 (9.8%)
Age (A)	
A0	20,725 (71%)
A1	8436 (29%)
Race (R)	
R1	23,308 (80%)
R2	4470 (15%)
R3	1383 (4.7%)

**Table 5:** Comparisons of C-indices by number of variables, data type, and method.

Number of Variables	Data Type	Method	C-Index	95%CI
5	Training	AJCC	0.7676	0.7622–0.7731
5	Training	EACCD	0.8293	0.8245–0.8341
5	Validation	EACCD	0.8437	0.8308–0.8566
7	Training	EACCD	0.8504	0.8461–0.8547
7	Validation	EACCD	0.8585	0.8468–0.8703

## Data Availability

The data presented in this study are available in the SEER database at https://seer.cancer.gov/data (accessed on 3 October 2023). A similarly structured source code written by Huan Wang used in this study is available in: https://github.com/hwang0113/Prognostic-System-for-Lymphoma (accessed on 4 June 2023). The source code was programmed and modified in R (Version 4.2.2) using the *survival* (Version 3.4-0), *cluster* (Version 2.1.4), *protoclust* (Version 1.6.4), *factoextra* (Version 1.0.7), and *compareC* (Version 1.3.2) libraries to generate the results of the EACCD studies in this manuscript.

## Supplementary Materials

The following supporting information can be downloaded at https://www.mdpi.com/article/10.3390/diagnostics15192462/s1, Table S1: Groups and their combinations in the five variable EACCD system. Table S2: Number of patients and events in each of the 10 groups in the five variable EACCD system. Table S3: Groups and their combinations in the seven variable EACCD system. Table S4: Number of patients and events in each of the 13 groups in the seven variable EACCD system.
