# Supplementary material for: Using Machine Learning to Create Prognostic Systems for Primary Prostate Cancer
Source: Diagnostics (Basel). 2025 Sep 26;15(19):2462. doi: 10.3390/diagnostics15192462 (PMC12523577; doi:10.3390/diagnostics15192462)
Supplement: Supplementary file 1 [file diagnostics-15-02462-s001.zip › diagnostics-3807949-supplementary.pdf]

Supplementary Table S1. Groups and their combinations in the five variable EACCD system.

**Group 1:**				
T1	N0	M0	P1	G1
T1	N0	M0	P1	G2
T1	N0	M0	P2	G1
T1	N0	M0	P2	G2
T1	N0	M0	P3	G1
T2a	N0	M0	P1	G1
T2a	N0	M0	P1	G2
T2a	N0	M0	P2	G1
T2a	N0	M0	P2	G2
T2a	N0	M0	P3	G1
T2a	N0	M0	P3	G2
T2bc	N0	M0	P1	G1
T2bc	N0	M0	P1	G2
T2bc	N0	M0	P2	G1
T2bc	N0	M0	P2	G2
T2bc	N0	M0	P2	G3
T2bc	N0	M0	P3	G1
T2bc	N0	M0	P3	G2
T2bc	N0	M0	P3	G3
T3	N0	M0	P2	G1
T3	N0	M0	P2	G2
T3	N0	M0	P3	G1
T3	N0	M0	P3	G2
**Group 2:**				
T1	N0	M0	P3	G2
T1	N0	M0	P3	G3
T2a	N0	M0	P3	G3
T2a	N0	M0	P3	G4
T2bc	N0	M0	P3	G4
T2bc	N1	M0	P3	G2
T2bc	N1	M0	P3	G3
T3	N0	M0	P2	G3
T3	N0	M0	P3	G3
**Group 3:**				
T1	N0	M0	P3	G4
T2a	N0	M0	P3	G5
T2bc	N0	M0	P2	G4
T2bc	N0	M0	P3	G5
T2bc	N1	M0	P3	G4
T3	N0	M0	P3	G4
T3	N1	M0	P3	G1
T3	N1	M0	P3	G2
T3	N1	M0	P3	G3
T4	N0	M0	P3	G1
T4	N0	M0	P3	G2
T4	N0	M0	P3	G3
**Group 4:**				
T1	N1	M0	P3	G2
T3	N0	M0	P3	G5
T3	N1	M0	P3	G4

**Group 5:**				
T1	N0	M0	P2	G4
T1	N0	M0	P3	G5
T1	N1	M0	P3	G4
T2bc	N1	M0	P3	G5
T3	N1	M0	P3	G5
T4	N1	M0	P3	G4
**Group 6:**				
T1	N1	M0	P3	G5
T3	N1	M1	P3	G4
T4	N0	M0	P3	G4
**Group 7:**				
T3	N0	M1	P3	G4
T4	N0	M0	P3	G5
T4	N1	M0	P3	G5
**Group 8:**				
T1	N0	M1	P3	G1
T1	N0	M1	P3	G2
T1	N0	M1	P3	G3
T1	N0	M1	P3	G4
T1	N1	M1	P3	G4
T2bc	N1	M1	P3	G4
**Group 9:**				
T1	N0	M1	P3	G5
T1	N1	M1	P3	G5
T2bc	N0	M1	P3	G4
T2bc	N0	M1	P3	G5
T2bc	N1	M1	P3	G5
T3	N0	M1	P3	G5
T3	N1	M1	P3	G5
T4	N0	M1	P3	G4
T4	N1	M1	P3	G4
**Group 10:**				
T4	N0	M1	P3	G5
T4	N1	M1	P3	G5

Supplementary Table S2. Number of patients and events in each of the 10 groups in the five variable EACCD system.

Group	N	Events
1	100341	1705
2	36367	1673
3	12559	1378
4	3070	621
5	4835	1170
6	265	110
7	381	180
8	846	497
9	2125	1549
10	423	336

Supplementary Table S3. Groups and their combinations in the seven variable EACCD system.

**Group 1:**						
T1	N0	M0	P1	G2	A0	R1
T1	N0	M0	P1	G2	A1	R1
T1	N0	M0	P2	G1	A0	R1
T1	N0	M0	P3	G1	A0	R1
T1	N0	M0	P3	G1	A0	R3
T2a	N0	M0	P1	G1	A0	R1
T2a	N0	M0	P1	G2	A0	R1
T2a	N0	M0	P2	G1	A0	R1
T2a	N0	M0	P2	G1	A1	R1
T2a	N0	M0	P2	G2	A0	R1
T2a	N0	M0	P2	G2	A1	R1
T2a	N0	M0	P3	G1	A0	R1
T2a	N0	M0	P3	G1	A0	R3
T2a	N0	M0	P3	G1	A1	R3
T2a	N0	M0	P3	G2	A0	R1
T2a	N0	M0	P3	G2	A0	R3
T2bc	N0	M0	P1	G1	A0	R1
T2bc	N0	M0	P1	G2	A0	R1
T2bc	N0	M0	P2	G1	A0	R1
T2bc	N0	M0	P2	G1	A0	R2
T2bc	N0	M0	P2	G1	A1	R1
T2bc	N0	M0	P3	G1	A0	R1
T2bc	N0	M0	P3	G1	A0	R2
T2bc	N0	M0	P3	G1	A0	R3
T2bc	N0	M0	P3	G1	A1	R2
T2bc	N0	M0	P3	G1	A1	R3
T2bc	N0	M0	P3	G2	A0	R1
T2bc	N0	M0	P3	G2	A0	R2
T2bc	N0	M0	P3	G2	A0	R3
T2bc	N0	M0	P3	G2	A1	R3
T3	N0	M0	P2	G1	A0	R1
T3	N0	M0	P2	G2	A0	R1
T3	N0	M0	P3	G1	A0	R1
T3	N0	M0	P3	G1	A0	R2
T3	N0	M0	P3	G1	A0	R3
T3	N0	M0	P3	G1	A1	R3
T3	N0	M0	P3	G3	A1	R3
**Group 2:**						
T1	N0	M0	P1	G1	A0	R1
T1	N0	M0	P1	G1	A1	R1
T1	N0	M0	P2	G1	A0	R2
T1	N0	M0	P2	G1	A1	R1
T1	N0	M0	P2	G2	A0	R1
T1	N0	M0	P2	G2	A1	R1
T1	N0	M0	P3	G1	A0	R2
T1	N0	M0	P3	G1	A1	R1

T1	N0	M0	P3	G1	A1	R2
T1	N0	M0	P3	G1	A1	R3
T1	N0	M0	P3	G2	A0	R1
T1	N0	M0	P3	G2	A0	R2
T1	N0	M0	P3	G2	A0	R3
T1	N0	M0	P3	G2	A1	R3
T1	N0	M0	P3	G3	A0	R3
T2a	N0	M0	P3	G1	A0	R2
T2a	N0	M0	P3	G1	A1	R1
T2a	N0	M0	P3	G2	A0	R2
T2a	N0	M0	P3	G2	A1	R1
T2a	N0	M0	P3	G3	A0	R1
T2a	N0	M0	P3	G3	A0	R3
T2bc	N0	M0	P2	G2	A0	R1
T2bc	N0	M0	P2	G2	A1	R1
T2bc	N0	M0	P2	G3	A0	R1
T2bc	N0	M0	P3	G1	A1	R1
T2bc	N0	M0	P3	G2	A1	R1
T2bc	N0	M0	P3	G3	A0	R1
T2bc	N0	M0	P3	G3	A0	R2
T2bc	N0	M0	P3	G3	A0	R3
T2bc	N0	M0	P3	G3	A1	R2
T2bc	N0	M0	P3	G4	A0	R1
T2bc	N0	M0	P3	G4	A0	R3
T3	N0	M0	P3	G1	A1	R1
T3	N0	M0	P3	G2	A0	R1
T3	N0	M0	P3	G2	A0	R2
T3	N0	M0	P3	G2	A0	R3
T3	N0	M0	P3	G2	A1	R3
**Group 3:**						
T1	N0	M0	P1	G1	A0	R2
T1	N0	M0	P2	G1	A1	R2
T1	N0	M0	P2	G2	A0	R2
T1	N0	M0	P3	G2	A1	R1
T1	N0	M0	P3	G2	A1	R2
T1	N0	M0	P3	G3	A0	R1
T1	N0	M0	P3	G3	A0	R2
T1	N0	M0	P3	G3	A1	R1
T1	N0	M0	P3	G3	A1	R3
T1	N0	M0	P3	G4	A0	R3
T2a	N0	M0	P3	G1	A1	R2
T2a	N0	M0	P3	G2	A1	R2
T2a	N0	M0	P3	G2	A1	R3
T2a	N0	M0	P3	G3	A0	R2
T2a	N0	M0	P3	G3	A1	R1
T2a	N0	M0	P3	G3	A1	R2
T2a	N0	M0	P3	G4	A0	R1
T2a	N0	M0	P3	G4	A0	R2
T2a	N0	M0	P3	G4	A0	R3
T2a	N0	M0	P3	G4	A1	R1
T2a	N0	M0	P3	G4	A1	R3
T2a	N0	M0	P3	G5	A0	R3
T2bc	N0	M0	P3	G2	A1	R2

T2bc	N0	M0	P3	G3	A1	R1
T2bc	N0	M0	P3	G3	A1	R3
T2bc	N0	M0	P3	G4	A0	R2
T2bc	N0	M0	P3	G4	A1	R3
T2bc	N0	M0	P3	G5	A0	R3
T2bc	N1	M0	P3	G2	A0	R1
T2bc	N1	M0	P3	G3	A0	R1
T3	N0	M0	P2	G3	A0	R1
T3	N0	M0	P3	G2	A1	R1
T3	N0	M0	P3	G3	A0	R1
T3	N0	M0	P3	G3	A0	R2
T3	N0	M0	P3	G3	A0	R3
T3	N0	M0	P3	G3	A1	R1
T3	N0	M0	P3	G4	A0	R2
T3	N0	M0	P3	G4	A0	R3
T3	N1	M0	P3	G2	A0	R1
**Group 4:**						
T1	N0	M0	P3	G3	A1	R2
T1	N0	M0	P3	G4	A0	R1
T1	N0	M0	P3	G4	A0	R2
T1	N0	M0	P3	G4	A1	R3
T1	N0	M0	P3	G5	A0	R3
T2a	N0	M0	P3	G3	A1	R3
T2a	N0	M0	P3	G4	A1	R2
T2a	N0	M0	P3	G5	A0	R1
T2bc	N0	M0	P2	G4	A0	R1
T2bc	N0	M0	P3	G4	A1	R1
T2bc	N0	M0	P3	G4	A1	R2
T2bc	N0	M0	P3	G5	A0	R1
T2bc	N0	M0	P3	G5	A0	R2
T3	N0	M0	P3	G4	A0	R1
T3	N0	M0	P3	G4	A1	R3
T3	N1	M0	P3	G2	A0	R2
T3	N1	M0	P3	G2	A1	R1
T3	N1	M0	P3	G3	A0	R2
T3	N1	M0	P3	G3	A1	R1
T4	N0	M0	P3	G2	A0	R1
**Group 5:**						
T1	N0	M0	P3	G4	A1	R1
T1	N0	M0	P3	G4	A1	R2
T2a	N0	M0	P3	G5	A0	R2
T2bc	N0	M0	P3	G5	A1	R3
T2bc	N1	M0	P3	G4	A0	R1
T3	N0	M0	P3	G3	A1	R2
T3	N0	M0	P3	G4	A1	R1
T3	N0	M0	P3	G5	A0	R3
T3	N1	M0	P3	G1	A0	R1
T3	N1	M0	P3	G3	A0	R1
T4	N0	M0	P3	G1	A0	R1
T4	N0	M0	P3	G3	A0	R1
**Group 6:**						
T1	N0	M0	P3	G5	A0	R1

T1	N0	M0	P3	G5	A0	R2
T1	N1	M0	P3	G2	A0	R1
T2a	N0	M0	P3	G5	A1	R1
T2bc	N0	M0	P3	G5	A1	R1
T2bc	N0	M0	P3	G5	A1	R2
T3	N0	M0	P3	G2	A1	R2
T3	N0	M0	P3	G4	A1	R2
T3	N0	M0	P3	G5	A0	R1
T3	N0	M0	P3	G5	A0	R2
T3	N0	M0	P3	G5	A1	R3
T3	N1	M0	P3	G4	A0	R1
T3	N1	M0	P3	G4	A0	R2
T3	N1	M0	P3	G4	A1	R1
T3	N1	M0	P3	G5	A0	R3
**Group 7:**						
T1	N0	M0	P2	G4	A1	R1
T1	N0	M0	P3	G5	A1	R1
T1	N0	M0	P3	G5	A1	R2
T1	N0	M0	P3	G5	A1	R3
T1	N1	M0	P3	G4	A0	R1
T1	N1	M0	P3	G4	A1	R1
T2a	N0	M0	P3	G5	A1	R2
T2bc	N1	M0	P3	G5	A0	R1
T2bc	N1	M0	P3	G5	A1	R1
T3	N0	M0	P3	G5	A1	R1
T3	N0	M0	P3	G5	A1	R2
T3	N1	M0	P3	G5	A0	R1
T3	N1	M0	P3	G5	A0	R2
T3	N1	M0	P3	G5	A1	R1
T4	N0	M0	P3	G4	A0	R1
T4	N0	M0	P3	G5	A0	R1
T4	N1	M0	P3	G4	A0	R1
**Group 8:**						
T1	N0	M1	P3	G2	A0	R1
T1	N1	M0	P3	G5	A0	R1
T1	N1	M0	P3	G5	A1	R1
T3	N0	M1	P3	G4	A0	R1
T3	N1	M1	P3	G4	A0	R1
T4	N0	M0	P3	G4	A1	R1
T4	N1	M0	P3	G5	A0	R1
**Group 9:**						
T1	N0	M1	P3	G1	A1	R1
T1	N0	M1	P3	G3	A0	R1
T1	N0	M1	P3	G3	A1	R1
T1	N0	M1	P3	G4	A0	R1
T1	N0	M1	P3	G4	A0	R2
T1	N0	M1	P3	G5	A1	R3
T1	N1	M1	P3	G4	A0	R1
T1	N1	M1	P3	G4	A1	R1
T2bc	N0	M1	P3	G4	A0	R1
T2bc	N0	M1	P3	G5	A0	R2
T2bc	N1	M1	P3	G4	A0	R1

T2bc	N1	M1	P3	G5	A1	R1
T4	N1	M0	P3	G5	A1	R1
**Group 10**						
T1	N0	M1	P3	G2	A1	R1
T1	N0	M1	P3	G4	A1	R1
T1	N0	M1	P3	G5	A0	R3
T2bc	N0	M1	P3	G5	A0	R1
T3	N0	M1	P3	G4	A1	R1
T4	N0	M0	P3	G5	A1	R1
**Group 11**						
T1	N0	M1	P3	G4	A1	R2
T1	N0	M1	P3	G5	A0	R1
T1	N1	M1	P3	G4	A0	R2
T1	N1	M1	P3	G5	A0	R1
T2bc	N1	M1	P3	G5	A0	R1
T3	N0	M1	P3	G5	A0	R1
T3	N1	M1	P3	G5	A0	R1
T4	N0	M1	P3	G4	A0	R1
T4	N0	M1	P3	G4	A1	R1
T4	N0	M1	P3	G5	A0	R1
T4	N1	M1	P3	G4	A0	R1
**Group 12**						
T1	N0	M1	P3	G5	A0	R2
T1	N0	M1	P3	G5	A1	R1
T1	N0	M1	P3	G5	A1	R2
T1	N1	M1	P3	G5	A0	R2
T1	N1	M1	P3	G5	A1	R1
T2bc	N0	M1	P3	G4	A1	R1
T2bc	N0	M1	P3	G5	A1	R1
T3	N0	M1	P3	G5	A1	R1
T3	N1	M1	P3	G5	A1	R1
T4	N1	M1	P3	G5	A0	R1
T4	N1	M1	P3	G5	A0	R2
**Group 13**						
T4	N0	M1	P3	G5	A1	R1
T4	N1	M1	P3	G5	A1	R1

Supplementary Table S4: Number of patients and events in each of the 13 groups in the seven variable EACCD system.

Group	N	Events
1	64549	644
2	47724	1357
3	23549	1277
4	7873	725
5	4673	588
6	4762	913
7	4159	1082
8	384	167
9	689	401
10	490	295
11	960	723
12	1210	891
13	190	156
